# Draft Genome Sequence of Spirochaeta sp. Strain JC202, an Endosymbiont of the Termite (Isoptera) Gut

**DOI:** 10.1128/genomeA.01481-14

**Published:** 2015-01-22

**Authors:** L. Tushar, T. Sravanthi, C. Sasikala, C. V. Ramana

**Affiliations:** a Department of Plant Sciences, School of Life Sciences, University of Hyderabad, P. O. Central University, Hyderabad, India; b Centre for Environment, Bacterial Discovery Laboratory, IST, JNT University, Hyderabad, Kukatpally Hyderabad, India

## Abstract

We announce here the draft genome sequence of Spirochaeta sp. strain JC202 isolated from gut of a termite (Isoptera). The genome suggests that Spirochaeta sp. JC202 has the capability for natural conjugation with the help of fimbriae and pili. Experimental evidence and the genome sequence suggest that strain JC202 is capable of producing colicin V and a bacteriocin group of peptides in a specific interaction.

## GENOME ANNOUNCEMENT

Members of the genus Spirochaeta are free living or live in association with insects and animals. The termite (Isoptera)-Spirochaeta association is a good example of symbiosis, in which the host (termite) provides shelter by creating an anaerobic niche and supplements the complex carbon (cellulose) required for the growth of Spirochaeta (1). The interactions of Spirochaeta with other bacterial systems has not studied to date, with the only exception of Spirochaeta-Clostridium enhancing cellulose degradation (2). Spirochaeta sp. strain JC202 was isolated from the termite gut and was grown in alkaline medium (3). Sequencing was carried out using Illumina Miseq 2 × 300-bp paired end chemistry. The sequence data were *de novo* assembled using the ABySS assembly software. Annotations were performed with 275 *de novo*-assembled contigs using the Rapid Annotations using Subsystems Technology (RAST) server (4).

The draft genome sequence of Spirochaeta sp. JC202 is 3,826,243 bp (3.82 Mb), with a G+C content of 59 mol%. The protein-coding bases total 3,190,883 bp, covering 83.39% of the total bases determined. The protein-coding genes of Spirochaeta sp*.* JC202 have an average length of 871.82 bases, ranging from 70 to 4,796 bases. Out of 3,660 open reading frames (ORFs) identified, 2,299 (62.81%) were functionally annotated, with 1,361 (37.18%) being hypothetical genes. The proposal of strain JC202 being a new species of the genus Spirochaeta is also evidenced from the species identification tool SpecI, which is based on core genome analysis, and the results support strain JC202 as a novel species (5).

Strain JC202 has a bacteroides aerotolerance operon (*batABDE*) and all the machinery to derive energy under anaerobic conditions (6). It has all the genes that are involved in periplasmic flagellar synthesis. The late competence protein *comEC* related to DNA transport is present in the genome, which suggests that the genome of strain JC202 is capable of natural competence (7). The draft genome of strain JC202 has around 11 genes involved in chitin and *N*-acetyl glucose amine utilization. Various stress response genes are present in the genome of strain JC202, which are involved mainly in oxidative, metal, temperature, and salt stress. The draft genome of strain JC202 has a HD-Gyp domain-containing gene, which is responsible for signaling and bacterial virulence to plants (8). Although the genomes of several spirochaetas have revealed the presence of genes for bacteriocins, these were never isolated and characterized.

The draft genome of strain JC202 has genes coding for phage terminase, prophage protein, phage capsid, scaffold Psp operon transcriptional activator, phage shock protein, hemagglutinin-like protein, prevent-host-death protein, mobile element protein, and phage peptidoglycan hydrolase. The presence of these viral genes in the genome of Spirochaeta sp. JC202 suggests that it is a carrier of prophage. Nuromedin U (*NmU*), coding for a peptide that stimulates the contraction of muscles in rats, is present in the genome of strain JC202, and this gene probably plays an important role in the elasticity of the cell (9).

### Nucleotide sequence accession numbers.

This whole-genome shotgun project has been deposited at DDBJ/EMBL/GenBank under the accession numbers JRAS00000000 and SRR1562012. The version described in this paper is version JRAS01000000.
